# Imbalanced target prediction with pattern discovery on clinical data repositories

**DOI:** 10.1186/s12911-017-0443-3

**Published:** 2017-04-20

**Authors:** Tak-Ming Chan, Yuxi Li, Choo-Chiap Chiau, Jane Zhu, Jie Jiang, Yong Huo

**Affiliations:** 1 Philips Research China - Health Systems, China, Philips Innovation Campus Shanghai, No. 1 Building, 10, Lane 888, Tian Lin Road, Shanghai, 200233 China; 2 Peking University First Hospital, Beijing, China

**Keywords:** Pattern discovery, Data mining, Prediction, Imbalanced data, Clinical data repository

## Abstract

**Background:**

Clinical data repositories (CDR) have great potential to improve outcome prediction and risk modeling. However, most clinical studies require careful study design, dedicated data collection efforts, and sophisticated modeling techniques before a hypothesis can be tested. We aim to bridge this gap, so that clinical domain users can perform first-hand prediction on existing repository data without complicated handling, and obtain insightful patterns of imbalanced targets for a formal study before it is conducted. We specifically target for interpretability for domain users where the model can be conveniently explained and applied in clinical practice.

**Methods:**

We propose an interpretable pattern model which is noise (missing) tolerant for practice data. To address the challenge of imbalanced targets of interest in clinical research, e.g., deaths less than a few percent, the geometric mean of sensitivity and specificity (G-mean) optimization criterion is employed, with which a simple but effective heuristic algorithm is developed.

**Results:**

We compared pattern discovery to clinically interpretable methods on two retrospective clinical datasets. They contain 14.9% deaths in 1 year in the thoracic dataset and 9.1% deaths in the cardiac dataset, respectively. In spite of the imbalance challenge shown on other methods, pattern discovery consistently shows competitive cross-validated prediction performance. Compared to logistic regression, Naïve Bayes, and decision tree, pattern discovery achieves statistically significant (*p*-values < 0.01, Wilcoxon signed rank test) favorable averaged testing G-means and F1-scores (harmonic mean of precision and sensitivity). Without requiring sophisticated technical processing of data and tweaking, the prediction performance of pattern discovery is consistently comparable to the best achievable performance.

**Conclusions:**

Pattern discovery has demonstrated to be robust and valuable for target prediction on existing clinical data repositories with imbalance and noise. The prediction results and interpretable patterns can provide insights in an agile and inexpensive way for the potential formal studies.

**Electronic supplementary material:**

The online version of this article (doi:10.1186/s12911-017-0443-3) contains supplementary material, which is available to authorized users.

## Background

### Data analytics on clinical data repositories

Healthcare Information Systems (HIS) such as Cardiovascular Information Systems (CVIS) have been available for decades [1]. The main function is to store and access patient records with deeper information than Electronic Medical Records (EMR). Integrated with EMR, Radiology Information Systems (RIS), Laboratory Information Systems (LIS), etc., HIS and CVIS have been useful for monitoring reporting, operating, scheduling and managing purposes with graphical user interfaces (GUI) such as dashboards.

With the emerging technology and availability of clinical registries and clinical data repositories [2], advanced predictive data analytics has great potential to add value to clinical research and improvement of clinical outcomes [3]. Traditional clinical studies, either retrospective or perspective, require tremendous efforts in design, data collection and sophisticated processing before any hypothesis can be tested or a target can be predicted. Mining existing massive practice data from repositories offers a promising way to create value and provide insights without too much extra overhead of a traditional clinical study. The challenge lies in the noises of practice data and imbalance of prediction targets of major clinical importance, such as bleeding after percutaneous coronary intervention (PCI) [4], or cardiac death [5]. Because data directly available from clinical data repositories is not subject to strict inclusion/exclusion criteria or sample matching to balance cases and controls [6], typical data mining methods for prediction (classification) are not designed to handle such challenges. The dilemma is that with rich existing data, domain users desire to generate initial data-driven hypotheses and get insights whether a specific clinical target of interest is predictable and what attributes (predictor variables) should be considered, before they take on the more involving way of a formal clinical study. As imbalanced target prediction is more challenging, it is also a realistically meaningful challenge which offers high practical value for outcome prediction and quality improvement in the real-world distribution of cases and control.

We aim to bridge the gap between sophisticated preparation to handle the imbalanced noisy data properties and first-hand data-driven insights for predicting targets of interest directly on existing data. In this regard, domain users can generate meaningful hypotheses and gain insights of their targets of interest with respect to predictability, and discover informative patterns of potential predictors to distinguish the targets from the others. The patterns discovered in this way are comparable to the best achievable discoveries requiring a series of sophisticated data processing, such as up-sampling with tweaking, before typical prediction methods can be applied. As a result, more involving clinical studies can be potentially guided for what data samples to include/exclude and what predictors (variables) to collect, for example, in a case report form (CRF) for a formal study.

### Model interpretability for domain users

Model interpretability is also highly desired in the clinical context for domain users. Technically speaking, the ability to draw classification boundaries on data is valid interpretability, but we specifically aim at clinical interpretability. In particular, it requires a prediction model can be explained by clinical domain practitioner, and applied, for example to select characteristics of patient cohorts that are expected to be consistent with the model predictability, to conduct his/her formal follow-up study. Therefore, interpretability throughout this paper represents a domain specific challenge rather than a technical one.

While domain users are gradually accepting more sophisticated prediction models in the clinical domains [7–13], we exclude the following models which are not considered domain interpretable in our scope: support vector machine (SVM) and artificial neural networks (ANN) which do not generate explainable rules for domain users [11, 14], random tree and random forest which generate an excessive number of trivial rules that are overwhelming for clinical reasoning [15]. For example, we test-ran random tree on the first real dataset in our evaluation, and it generated a tree that spans 228 lines (attribute-value occurrences). Though technically it can be considered as a decision tree, the lengthy rules are not feasibly interpretable for domain users.

In order to identify predictive patterns to guide potential formal studies, interpretability is critical for not only the selection of attributes (predictors) but also the specific properties (values) of the predictors to look into. Therefore, among the numerous classification methods available, we focus on interpretable ones in our proposed method and comparison, which should include the explicit attributes and values in the trained models (classifiers) with a model length digestible by human users. The representable interpretable models included in our evaluation comparisons are logistic regression [16], Naïve Bayes [17] and decision tree [18].

In this paper, we propose a predictive and intuitively interpretable pattern model that is noise tolerant for real data. We develop a simple pattern discovery algorithm where an optimization criterion is employed for prediction targets that are rare but of clinical importance, such as cardiac death. To evaluate the effectiveness, we employed two retrospective clinical datasets with imbalance and compared pattern discovery with the above representative interpretable prediction methods. Evaluation with cross-validation shows competitive prediction performance of pattern discovery. Pattern discovery is expected to be a handy and valuable analytics tool for domain users to predict imbalanced targets from existing practice data without sophisticated processing, and to provide first-hand insights for formal research and studies to follow.

### Problem definition and related works

In this section, we define the problem we address and review the key related works. Data mining has been extensively applied in healthcare domain, which is believed to be able to uncover new biomedical and healthcare knowledge for clinical and administrative decision making as well as generate scientific hypotheses [3]. We focus on the prediction problem of classification, where for a given (training) dataset D, we would like to utilize the known (labelled) values of a target T to establish (train) a model and method (a classifier) to predict a target of interest (T = t), i.e. positive cases, for future (testing) data where T is not known. Specifically, the dataset

$$ \mathrm{D}=\left[\begin{array}{c}\hfill {D}_1\hfill \\ {}\hfill \vdots \hfill \\ {}\hfill {D}_n\hfill \end{array}\right]=\left[\begin{array}{c}\hfill {d}_{11}\hfill \\ {}\hfill \vdots \hfill \\ {}\hfill {d}_{n1}\hfill \end{array}\begin{array}{c}\hfill \cdots \hfill \\ {}\hfill \ddots \hfill \\ {}\hfill \cdots \hfill \end{array}\begin{array}{c}\hfill {d}_{1 m\hbox{'}}{t}_1\hfill \\ {}\hfill \vdots \hfill \\ {}\hfill {d}_{n m\hbox{'}}{t}_n\hfill \end{array}\right]=\left[{R}_1,{R}_2\dots, {R}_m,\mathrm{T}\right] $$

is with n samples and m + 1 attributes (columns) where for simplicity the first m attributes R = [R_1_, R_2_, … R_m_] represent the predictor variables and the last attribute T represents the target to predict (response). d_ij_ is a value in D for attribute R_i_ for i = 1, 2, …, n and j = 1, 2, …, m. T is a nominal attribute and one is specifically interested in cases of T = t, compared to cases of any other values. Therefore, we model the problem as binary classification where we would like to distinguish T = t (positive) from T ≠ t (negative, and can be of multiple values in data). We assume there are no missing values of T in training, but R can have certain missing values, reflecting the reality of healthcare data in practice. Furthermore, most targets of clinical interest (T = t) are minorities in real data, e.g. Cardiac death = Yes and Death in 1 year = Yes. In such a case, the prevalence, defined as # (T = t)/n, is considerably smaller than 1/2 (50%), and we interchangeably denote the dataset and prediction problem as imbalanced.

We have listed existing interpretable classifiers included for comparisons: logistic regression, Naïve Bayes, and decision tree (C4.5). They were not designed for imbalanced datasets. Naive Bayes would be less influenced as the target proportion could be used as the prior in training. But a moderately high imbalance ratio would overweigh the prior and impact the prediction performance, as will be shown in experimental results and recent work [13]. Both logistic regression and decision tree optimize towards the overall accuracy where the prediction performance of a minority target can be significantly influenced.

The other non-interpretable methods, such as k-nearest-neighbor [19], support vector machines [20] and artificial neural nets [3], are beyond our scope of comparison as they do not directly provide explicit human-readable “patterns” to follow up for domain users.

The proposed pattern discovery in this work has some resemblance with association rule mining [21], associated motif discovery from biological sequences [22] and feature selection for data mining [23]. Association rule finds only frequent items, but does not model prediction (classification). One critical limitation of association rule based methods is that the target has to be frequent, which is not the case in clinical outcomes of interest [6]. Further extensions of classification after association rule mining suffer from scalability because non-trivial rules (over 3 attributes) can take intractable time to compute [24]. Furthermore, association rule mining works with only exact occurrences which cannot tolerate noises in healthcare data. These two limitations also apply to rule extraction based prediction methods [25]. Motif discovery works on sequential and contiguous patterns which are not the case in mining healthcare data (attributes are disjoint without an order and are not contiguous) [22, 26]. Nonetheless, the approximate matching modeling of biological motifs [27] inspires us to introduce a control to tolerate noise and increase flexibility of the pattern model. Feature selection usually works as an auxiliary method in combination with formal data mining methods for target prediction [23], but it works only on the attribute level (not attribute-value) and does not explicitly generates an prediction model for direct interpretation. On the other hand, the wide spectrum of feature selection methods provides many choices to select attributes for pattern discovery, such as Chi-Squared test based feature selection [28].

Motivated by these, this work presents a pattern discovery classifier featuring a highly interpretable predictive pattern model on noisy, imbalanced healthcare data in practice for domain users.

## Methods

### Data

In this study, we utilize two published datasets to evaluate how pattern discovery can be applied on imbalanced target prediction, similarly in the way for clinical data repositories where minimum data processing is needed. The two datasets have been de-identified and published online for scientific research. The availability and approval information can be found from the corresponding references.

The thoracic dataset is about surgical risk for real-life clinical data from the thoracic surgery domain. The data was originally collected retrospectively at Wroclaw Thoracic Surgery Centre for patients who underwent major lung resections for primary lung cancer in the years 2007–2011 [20]. The publicly available dataset is after feature selection and elimination of missing values. It is composed of 470 samples, 16 pre-operative attributes after feature selection, and the target attribute of 1-year survival period labels (denoted as Risk1Yr = Yes if patient died; prevalence = 14.9%). To simulate the target scenario without requiring much tweaking, the original numeric attributes (PRE4, PRE5, and age) without well-established categorization were skipped, the total 22 missing values (0.3%) in the data were kept as-is and no imputation was done to evaluate noise handling. Instead, PRE4 and PRE5 were combined into the well-established chronic obstructive pulmonary disease COPD (Yes/No) category with the auxiliary function. The attribute list is detailed in the Additional file 1.

The cardiac death dataset contains patients with coronary artery disease (CAD). Peripheral blood samples from 338 subjects aged 62 ± 11 years with CAD were analyzed, and followed for a mean 2.4 years for cardiovascular death (31 deaths). The available dataset is composed of 43 attributes (41 non-trivial) covering both clinical attributes and derived ones from gene expressions [5]. While the study discovers association between gene expression profiles and cardiac death, the next question of both great interest and challenge to domain users is whether a predictive pattern can be discovered for more follow-up studies. Therefore, in the experiments we tried the prediction of Cardiac Death = Yes (prevalence = 9.1%) on the available data as-is, with the definition dependent removed to properly evaluate the prediction performance. In our experiments, data of both phases was combined for evaluation. In this dataset, gene expressions were transformed into more concise principal components (Prin*), and conserved axes of variation (snmAxis*). In our experiments, the gene expression components/axes were categorized by their signs (>0 or ≤ 0) with the auxiliary function. Other clinical indicator attributes were categorized according to typical normal/abnormal ranges. The total 417 missing values (2.9%) were kept to evaluate noise handling. The attribute list and more details on categorization are available in the Additional file 1.

### Pattern discovery

We first propose the pattern model to support interpretability and tolerate noise for real data. An optimization criterion for prediction performance on imbalanced targets is then employed. A simple algorithm is then presented to computationally discover a predictive pattern according to the optimization goal.

The proposed model is a combination of attributes and their corresponding (categorized) values for a chosen prediction target. An auxiliary configuration function is implemented to transform numeric values to categories according based on clinical guidelines or domain knowledge. To make the pattern practical and flexible for noisy realistic data, a matching ratio threshold is introduced. It controls the minimal percentage of attribute-value pairs to match where a sample can be considered an imperfect match of the pattern.

A pattern is proposed to be a selection of attributes and their corresponding values of a chosen target of interest, which is a selected attribute and its value to predict (T = t). A pattern is further proposed to be associated with matching (ratio) threshold *r*, requiring a minimal ratio *r* of attribute-values to match for a record to be considered as matched by a pattern. A pattern (Pat) is formally defined as {P, S, r}, which consists of

1. a subset of attributes P = {P_1_, P_2_, …, P_w_} ⊂ R,
2. a specific set of their corresponding values S = {v_1_, v_2_, …, v_w_}, and
3. a matching ratio threshold 0 < r < = 1 to control the ratio of matching values of a data sample on P.

It can be also represented as P_1_ = v_1_, P_2_ = v_2_, …, P_w_ = v_w_ (with matching ratio threshold = r)

Pattern matching: a sample D_i_ = {d_i1_, d_i2_, …, d_im_} is defined to match a pattern Pat = {P, S, r} = {{ P_1_, P_2_, …, P_w_ }, {v_1_, v_2_, …, v_w_}, r}, if count(d_iP1_ == v_1_, d_iP2_ == v_2_, …, d_iPw_ == v_w_)/m > = r. We denote this case as *match*(Pat, D_i_) = TRUE. Otherwise *match*(Pat, D_i_) = FALSE.

A simple illustrative dataset is presented in Table 1. There are 6 samples and 6 attributes excluding ID (irrelevant in prediction), where the target is Bleeding = Yes. Prevalence = 2/6 = 33%, and positive/negative ratio = 1/2 = 0.5.

**Table 1:** An illustrative example of categorical CVIS patient data

ID	Gender	PCI History	Hemoglobin	Diabetes	CRP	Bleeding
1	Male	Yes	Abnormal	No	Abnormal	Yes
2	Female	No	Abnormal	N/A	Abnormal	No
3	Male	No	N/A	No	Normal	No
4	N/A	Yes	Normal	No	Normal	No
5	Female	Yes	N/A	No	Abnormal	Yes
6	Male	No	Normal	No	Normal	No

*N/A* Not available (missing value), *PCI* Percutaneous coronary intervention, *CRP* C-Reactive Protein

The following example shows a candidate pattern to be discovered for target Bleeding = Yes

PCI History = Yes, Hemoglobin = Abnormal, CRP = Abnormal

Matching ratio threshold *r* = 2/3 (at least 2 attributes to match; or presented in % as 67%)

The threshold *r* thus tolerates missing values by allowing them as mismatches. Therefore, the pattern model is intuitively interpretable by clinical users. The challenge is about discovering a pattern computationally from data that maximizes certain prediction criterion *de novo*.

To optimize and evaluate the pattern model specifically on imbalance target, the following criteria are employed.

For a dataset D with m attributes R = {R_1_, R_2_, …, R_m_}, there are an exponential number of attribute-value combinations as pattern candidates, so we need certain optimization criterion to distinguish informative candidates from spurious ones. For the imbalanced minority target of interest T = t, the prediction performance should be evaluated by criteria other than accuracy, as it is non-informatively high (=1-prevalence) if one simply predicts all samples to be the majority cases T ≠ t.

Specifically, a classifier (pattern) can be evaluated by precision (pre) and sensitivity (sen) on predicting the minority target T = t when the labels of T can be obtained [29]. To collectively evaluate prediction performance, F1-score is usually employed which summarizes both by their harmonic mean (F1-score = 0 if number of true positive TP = 0) [29]:

$$ \mathrm{F}1\hbox{-} \mathrm{score}\kern0.5em =2\kern0.5em \ast \kern0.5em \mathrm{p}\mathrm{r}\mathrm{e}\kern0.5em \ast \kern0.5em \mathrm{s}\mathrm{e}\mathrm{n}/\left(\mathrm{pre}\kern0.5em +\kern0.5em \mathrm{se}\mathrm{n}\right) $$

Similarly, specificity (spec) can be calculated. A similar evaluation measure is G-mean, defined as the geometric mean of sensitivity and specificity:

$$ \mathrm{G}\hbox{-} \mathrm{mean}\kern0.5em =\sqrt{\mathrm{sen}\kern0.5em \ast \kern0.5em \mathrm{spec}} $$

All these measures have the range [0, 1] and are higher the better towards the ideal value 1. The evaluation steps of the candidate pattern on the illustrative data are shown in the Additional file 1.

These evaluation measures therefore serve as potential optimization criteria for a classifier targeting the prediction of minority T = t. In this work, we employ G-mean as the optimization criterion, which shows stronger trends for performance balance than F1-score in optimization (geometric mean versus harmonic mean) in initial experiments (details not shown). The optimization of G-mean is only carried out on training data, not on testing data.

The pattern discovery problem can be therefore defined as: given an input dataset D with input attributes R = {R_1_, R_2_, … R_m_} and target attribute T, a specified target of interest T = t, and a maximal pattern width W (< = m), find a pattern Pat = {P, S, r} where P ⊂ R, |P| < = W, such that the optimization criterion of G-mean for T = t is maximized on D.

The next challenge is to discover a pattern *de novo* to maximize the optimization criterion on the training data. We introduce a simple pattern discovery algorithm and further integrate it with independent log likelihoods for cases with too weak patterns to form the pattern discovery classifier.

For pattern discovery, search exhaustively is computationally intractable. The search space can be broken down into three steps in a simplified view: the candidate attributes; the optimal combination of possible values of the attributes; and the optimal matching threshold. The first two steps are still computationally intractable to reach optimal solutions with respect to measures such as f-measure [23]. A heuristic computational method is developed to discover a feasible pattern candidate first by eliminating hundreds of thousands of less predictive candidates, so that clinical users can have a feasible pattern to start with during interactions.

Identifying pattern candidate attributes is a feature selection problem [23]. The Chi-squared test of independence [28] is employed, which is well established and interpretable for domain users. To determine pattern width W, a cutoff of p-value (<= 0.05), or top K significant attributes can be used.

To tackle the challenge of determining the attribute-value combinations for imbalanced target prediction, we develop a heuristic method based on attribute-value percentage comparison. For a candidate attribute, all its values are listed with the target value (T = t) and non-target value (T ≠ t) in a table. The count of samples belonging to each specific attribute-target value combination is filled in. The row-wise percentages are then calculated. The heuristic method then compares these percentages column-wise and selects the value with the maximal percentage to associate with the target value. An illustrative example is shown in the Additional file 1.

Lastly, the matching ratio threshold *r* is determined from the exhaustive range of at least one attribute (1/W) up to all attributes (W/W = 100%), where the value generating the best optimization criterion is chosen as the output *r*.

Though the pattern model is intuitively interpretable, there can be cases with too weak and ambiguous patterns to discover when imbalance exists. To construct a robust classifier not to miss a case like this, we calculate the log likelihood of T = t with the attribute-values of the case along the pattern, and accepts cases if the log likelihood is larger than T ≠ t. This intuitively integrates the Naïve Bayes scoring to classify cases without any explicit patterns. We set a relatively loose criterion of positive/negative ratio < 2 to trigger the log likelihood scoring. The setting is for use convenience, as it is intuitively the minimal integer > 1, which is the boundary case of balanced data. Further optimizing this with decimal points may improve the results but it is not our current focus.

The training and classifying procedures of the pattern discovery classifier are summarized as follows:

#### Train classifier on training set D

Chi-squared test to select W attributes (W specified by user or by *p*-value cutoff): P = {P_1_, P_2_, …, P_w_} ⊂ R

Heuristic method to find values with the maximal row-wise percentages across the columns for the attributes: S = {v_1_, v_2_, …, v_w_} for T = t

For *r* = 1/W to W/W

Evaluate {P, S, *r*} on D and keep the pattern Pat with the best G-mean

Calculate the log likelihoods for all values of P = {P_1_, P_2_, …, P_w_} if imbalance exists

#### Classify D_i_ in a test set

Return *match*(Pat, D_i_) || (log likelihood (T = t |D_i_) > log likelihood (T ≠ t | D_i_) if calculated)

### Evaluation methods and experiment design

**Fig. 1:**
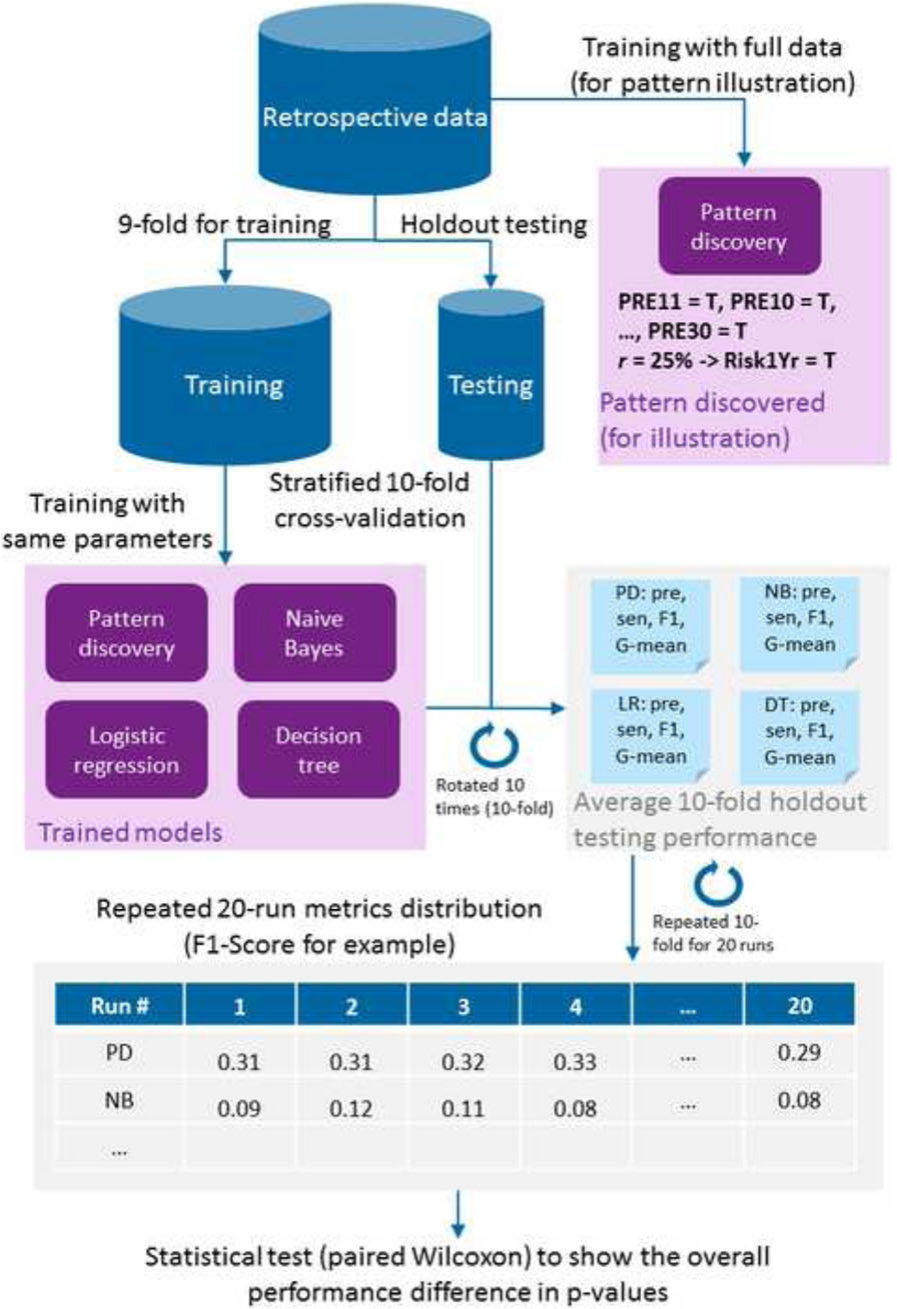
The evaluation framework designed for the experiments

In this sub-section, we illustrate the evaluation methods and experiment design for the results section. The whole evaluation framework designed for the experiments is illustrated in Fig. 1.

To evaluate prediction performance, a typical way is to use holdout testing data after building prediction models on training data. The training data could be further split to optimize parameters and select the model with the best generality before testing by applying cross-validation [30]. In this work, our aim is to evaluate model prediction generality for domain users with minimal tweaking and the datasets are retrospective ones. Instead of using one-off training-testing split which may introduce bias, we repeated training-testing multiple times (10) and recorded the average holdout testing performance each time. This was effectively a stratified 10-fold cross-validation, but without optimizing parameters or selecting top models. We further performed this rotated 10-time holdout testing 20 runs, resulting in a distribution for each prediction performance metrics of precision, sensitivity, F1-score and G-mean. Besides comparing the averaged 20-run metrics along with their standard deviations (±), we further evaluated the statistical significance of the performance distributions, as illustrated at the bottom of Fig. 1.

The non-parametric paired Wilcoxon signed rank test was applied [31], to assess whether the favorable (higher) F1-scores and G-means of pattern discovery were statistically significant compared to other method. We used R (Version 3.2.1) to perform this evaluation, particularly wilcox.test() with the following parameters: *paired = T, alternative = ‘greater’*.

A 10-fold cross-validation used in this training-testing way would generate 10 (slightly) different models due to the holdout difference. It is tricky to list all models of the 20 runs or synthesizing a unified one. We employed the common practice for illustration on retrospective data [32], which is to use full data to train a final model, also consistent with the way of rules illustrated in the thoracic dataset reference [20]. Note that in this regards the discovered pattern would be for illustration simplicity only, and a future testing set should be used to validate it. The final pattern generation part is illustrated in top right of Fig. 1.

The methods compared, including logistic regression, naive Bayes, and decision tree (C4.5), were run with the Weka 3.6 APIs which was able to run over missing values [32]. A random baseline classifier with equal chance to predict positive/negative for any sample was implemented, serving as a non-informative random guess method. This method has the theoretical sensitivity = 0.5 and precision = prevalence for any specified target. Therefore, no standard deviation is available. The Weka APIs of evaluation were employed to compute the metrics for all methods. All methods were run with the default parameters on the same set of attributes. Therefore the cross-validation was for evaluating the holdout testing each time rather than parameter optimization. Note that all models were trained on the same generated folds in each run for fair comparisons.

Targeting for the domain user scenarios, we focus on performance evaluation with the original prevalence (imbalance) of data. On the other hand, we notice that there are specific methods on down-sampling [11], up-sampling [10], or generating new artificial samples (such as synthetic minority over-sampling technique: SMOTE [33]) to address imbalanced data besides the typical cost matrix (high penalty on misclassified target cases in training) approaches [34]. While they have yielded promising results in many other applications, in our target scenario to gain initial insights from practice data, clinical domain users would be confused and disengaged by the statistics not reflecting the real data, either linked to non-existing samples or prevented from viewing certain real samples. This would become a concern beyond the scope here as we aim to provide interpretability for domain users to investigate into and connect to the actual samples.

Nevertheless, we performed extended experiments with up-sampling. We used the same evaluation framework, where additionally we up-sampled the minority positive cases to certain positive/negative ratios (up-sampling ratios) in the training set only, and evaluated the holdout testing set WITHOUT any up-sampling. Note that our purpose is to illustrate that pattern discovery can achieve comparably robust performance with the original imbalanced prevalence. This was done not for the scenario desired by healthcare domain users, as interpretability would be affected with non-existing samples and distorted case proportions.

## Results

### Results on the thoracic dataset

Following the experiment design, we first report the average 20-run testing results on the thoracic dataset with the original prevalence. Then we cover the extended experiment results with up-sampling. Statistical test results are then summarized, and the discovered pattern is illustrated with references to results beyond our scope. Detailed evaluation results with standard deviations (±) are available in the Additional file 1.

**Fig. 2:**
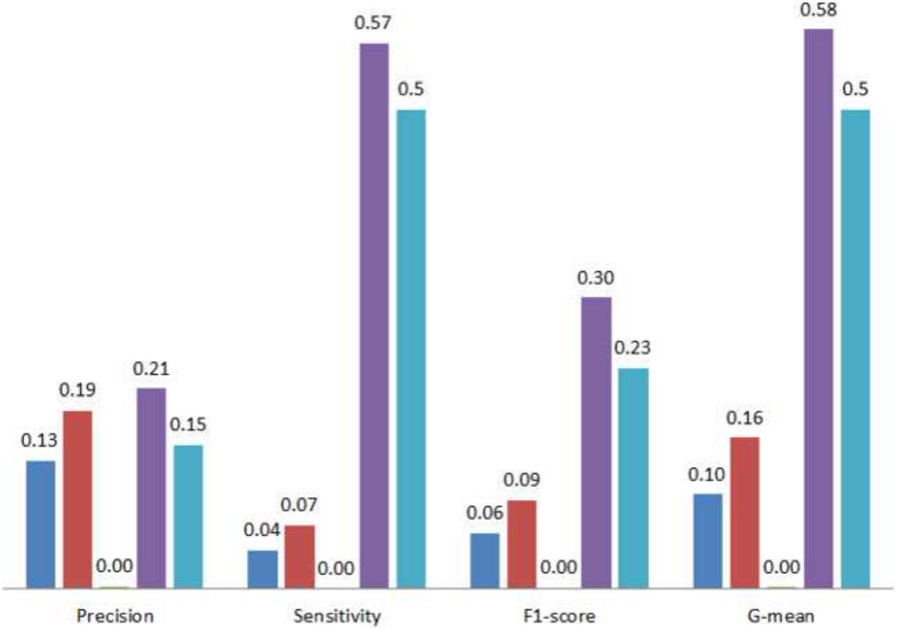
Average testing performance from 20-run 10-fold cross-validation on Risk1Yr = Yes of the thoracic dataset, with the original prevalence. Legends: logistic regression (*blue*), naive Bayes (*red*), decision tree (*green*), pattern discovery (*purple*), and random baseline (*light blue*)

The average precision, sensitivity, F1-score and G-mean of the methods compared are shown in Fig. 2. It may look surprising that except pattern discovery, the other methods perform even worse than the random baseline. Logistic regression and naive Bayes show poor prediction performance, resulting in 0.06 ± 0.02 and 0.09 ± 0.02, respectively on F1-score. Decision tree almost misclassifies all testing cases into the majority (0.00 ± 0.01). In this challenging setting (original prevalence = 14.9%), only pattern discovery is able to achieve non-trivial favorable prediction performance in all measures against the random baseline (e.g. F1-score 0.30 ± 0.01 vs 0.23 ± no standard deviation). It is likely that logistic regression optimizes the loss function of accuracy which is dominated by the majority. Naive Bayes is less influenced than logistic regression with the target prior. However, due to the imbalance, all methods except pattern discovery achieve very low sensitivity.

**Fig. 3:**
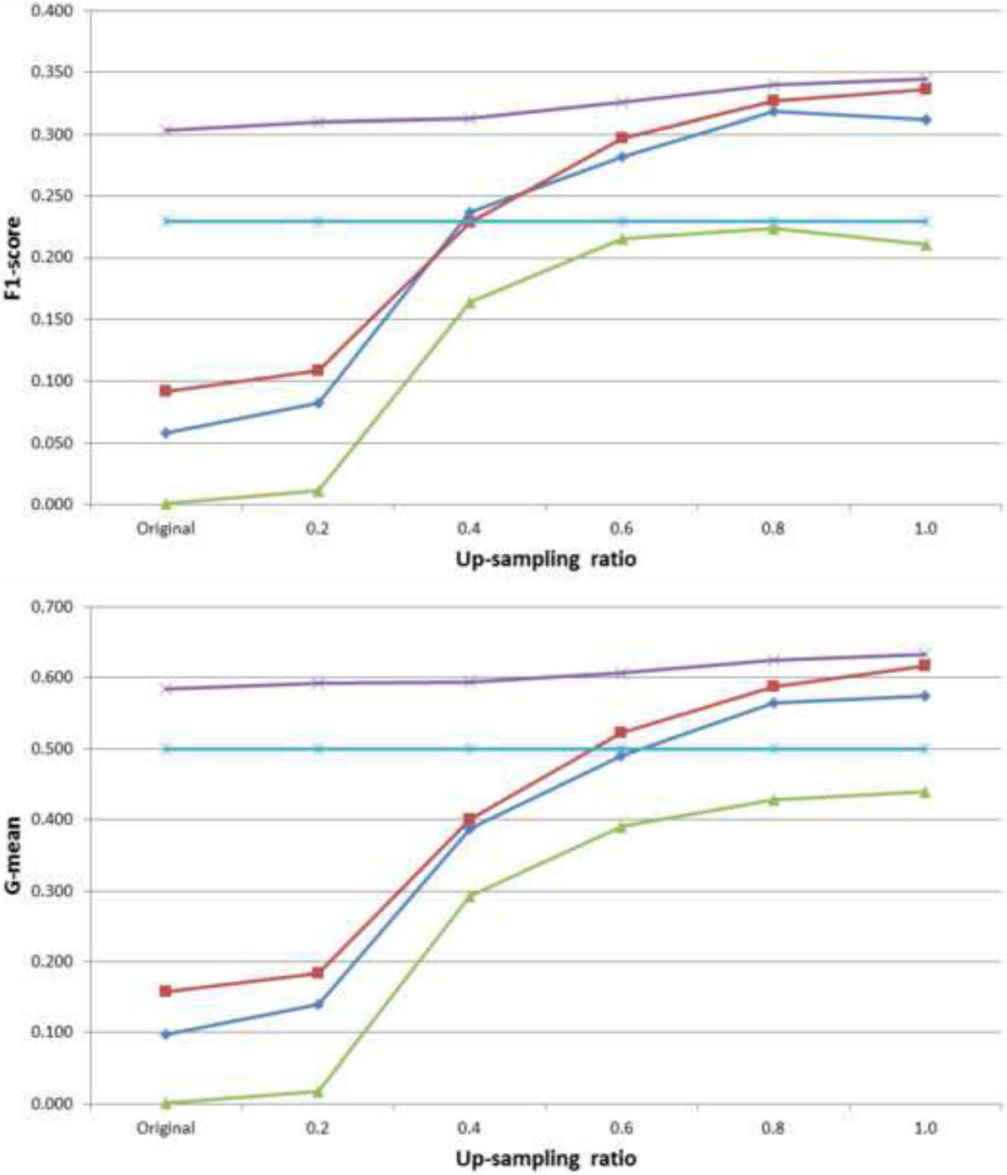
Average testing F1-scores (*top*) and G-means (*bottom*) from 20-run 10-fold cross-validation on Risk1Yr = Yes, with original prevalence and different up-sampling ratios (x-axes). Legends: logistic regression (*blue diamonds*), naive Bayes (*red squares*), decision tree (*green triangles*), pattern discovery (*purple crosses*), and random baseline (*light blue asterisks*)

By the extended up-sampling experiments, we demonstrate that the performance difference is mainly due to imbalance which is handled well by pattern discovery. The original positive/negative ratio was 0.18 corresponding to prevalence 14.9%; an up-sampling ratio up to 1.0 indicates no imbalance. As shown consistently in Fig. 3, pattern discovery has achieved very competitive average testing F1-score (0.30 ± 0.01) and G-mean (0.58 ± 0.01) without requiring any up-sampling, while the other methods are only able to achieve non-trivial results with substantial up-sampling up towards ratio 1.0. Till up-sampling ratio 1.0 does naive Bayes achieve the average F1-score (0.34 ± 0.02) almost as high as pattern discovery’s (0.35 ± 0.01), and G-mean (0.62 ± 0.02) in a similar manner (compared to pattern discovery’s 0.63 ± 0.01). Without any up-sampling, pattern discovery shows robust and favorable prediction performance to all other methods. We also have some investigation into higher up-sampling ratios beyond the valid imbalance assumption, and the results, available in the Additional file 1, consistently support our conclusion.

Therefore, finding the optimal up-sampling ratios beforehand is not trivial especially for domain users. Pattern discovery shows the advantage of robust and consistent prediction performance even without up-sampling, being the least sensitive to training up-sampling ratios. This is desirable for our target scenario where domain users would like to discover insights from noisy and imbalanced practice data before they further invest heavily into formal studies.

For both F1-scores and G-means with the original prevalence, pattern discovery has already shown statistically significance (*p*-value = 4.43 × 10^−5^) over the random baseline which has fixed results for all different up-sampling ratios, so we focus on detailed comparison with other methods for simplicity. As shown in Table 2, pattern discovery has shown clear statistical significance (significance level 0.01) at all up-sampling ratios except for F1-score compared to naive Bayes at 1.0 (*p*-value = 0.0895).

**Table 2:** Wilcoxon test (paired, greater than) *p*-values between pattern discovery and the other methods on testing F1-scores and G-means of the cross validations on the thoracic dataset

	Logistic Regression	Naive Bayes	Decision Tree
F1-score			
Original	4.43 × 10^−5^	4.43 × 10^−5^	4.43 × 10^−5^
0.2	4.43 × 10^−5^	4.43 × 10^−5^	4.43 × 10^−5^
0.4	4.43 × 10^−5^	4.43 × 10^−5^	4.43 × 10^−5^
0.6	4.43 × 10^−5^	8.14 × 10^−5^	4.43 × 10^−5^
0.8	7.54 × 10^−4^	0.0045	4.43 × 10^−5^
1.0	5.17 × 10^−5^	0.0895	4.43 × 10^−5^
G-mean			
Original	4.43 × 10^−5^	4.43 × 10^−5^	4.43 × 10^−5^
0.2	4.43 × 10^−5^	4.43 × 10^−5^	4.43 × 10^−5^
0.4	4.43 × 10^−5^	4.43 × 10^−5^	4.43 × 10^−5^
0.6	4.43 × 10^−5^	4.43 × 10^−5^	4.43 × 10^−5^
0.8	4.43 × 10^−5^	4.43 × 10^−5^	4.43 × 10^−5^
1.0	4.43 × 10^−5^	9.72 × 10^−4^	4.43 × 10^−5^

*p-value = 4.43 × 10^−5^ indicates higher rankings of paired values in all 20 runs for pattern discovery

For illustration, we show the patterns discovered with all data on for cardiac Risk1Yr = Yes (in the data T means Yes and F means No).

Without using the numeric attributes (including PRE4, PRE5, and age) same as in the cross-validation, the pattern of 12 categorized attributes achieves F1-score 0.337 (without up-sampling) as shown in Table 3, with coverage and accuracy shown for reference only.

**Table 3:** Discovered pattern from full thoracic dataset for illustration

Pattern (Rule)	Coverage	Accuracy
PRE11 = T, PRE10 = T, PRE9 = T, PRE8 = T, PRE7 = T, PRE6 = PRZ2, COPD = Yes, PRE25 = T, DGN = DGN5, PRE17 = T, PRE14 = OC14, PRE30 = T; *r* = 25% - > Risk1Yr = T	0.42	0.23
OTHERWISE - > Risk1Yr = F	0.58	0.91

Although our focus is on interpretable models and minimal sampling handling to fit the target scenario for domain users, we are aware that advanced sampling combined with non-interpretable methods such as support vector machine (SVM) could generate very promising prediction performance [10, 11, 33], with also reported evaluation results on this dataset [20]. We listed the reference G-mean and projected the F1-score with pattern discovery’s results generated at up-sampling ratio 1.0, solely for audience information. Note that this is not a formal comparison as the methods in the list were not interpretable methods in our scope, where the referenced cross-validation experiment was not with the same fold or run numbers.

As listed in Table 4, pattern discovery, with a naturally interpretable model and without sophisticated sampling techniques, is able to provide very close prediction performance to the top reported results (0.03 and 0.024 differences from the top F1-score and G-mean, respectively). As mentioned in the reference, boosted SVM for imbalanced data (BSI) is highly uninterpretable for clinical practitioners because it combines SVM and ensembles [20]. JRip + BSI shows a non-trivial effort to extract interpretable rules (with JRip [25]). Nonetheless, in the target scenario for domain users, pattern discovery shows its unique value and convenience, compared to sophisticated processing which probably further requires careful tweaking.

**Table 4:** F1-scores and G-means of pattern discovery and the referenced non-interpretable methods

Methods	F1-score	G-mean
pattern discovery	*0.345* ^a^	*0.633* ^a^
RUSBoost (RUS) [10]	0.302	0.588
SVM + SMOTE (SSVM)	0.338	0.625
boosted SVM for imbalanced data (BSI)	0.375	0.657
JRip + BSI	0.362	0.648
UnderBagging (UB)	0.354	0.651

^a^Average testing results from our experiment; other results reported or projected from the reference [20]

Further categorizing on PRE4, PRE5, and age, we are able to get a pattern with F1-score 0.41 (precision 0.40, sensitivity 0.42; including AGE > = 80 and PRE5 < = 3.62) comparable to 0.44 by the JRip + BSI rules. Compared to the 9 rules extracted from JRip + BSI that are complex and less handy for practice (details in reference [20], also available from the Additional file 1), our discovered pattern is concise and practically interpretable for domain users, demonstrating its value to be used in the target scenario. The results shows great potential when pattern discovery is fully utilized with domain knowledge.

### Results on the cardiac death dataset

This sub-section reports the same experiment results and comparisons on the cardiac death dataset.

**Fig. 4:**
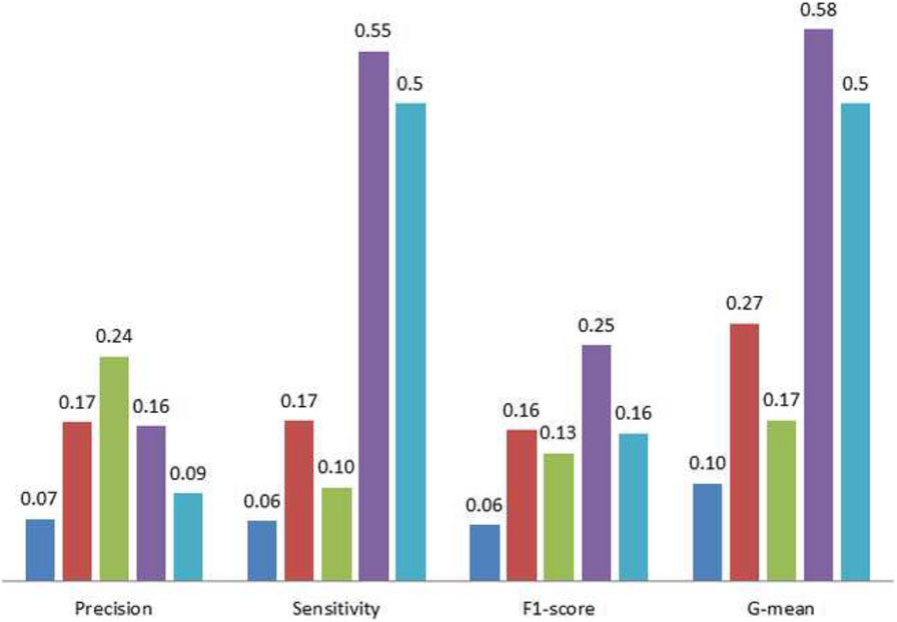
Average testing performance from 20-run 10-fold cross-validation on Cardiac death = Yes with the original prevalence. Legends: logistic regression (*blue*), naive Bayes (*red*), decision tree (*green*), pattern discovery (*purple*), and random baseline (*light blue*)

As shown in Fig. 4 with the original prevalence, pattern discovery again demonstrates robust prediction performance with comparable precision, the highest sensitivity, reaching non-trivial F1-score (0.25 ± 0.03) and G-mean (0.58 ± 0.05) compared to other methods and the random baseline. Note that pattern discovery also has the lowest standard deviations compared the other methods on both F1-score and G-mean. While decision tree is able to achieve the best precision, the low sensitivity contributes to the overall low F1-score and G-mean. The relative rankings of logistic regression, naive Bayes, pattern discovery and the baseline are unchanged compared to Fig. 2.

**Fig. 5:**
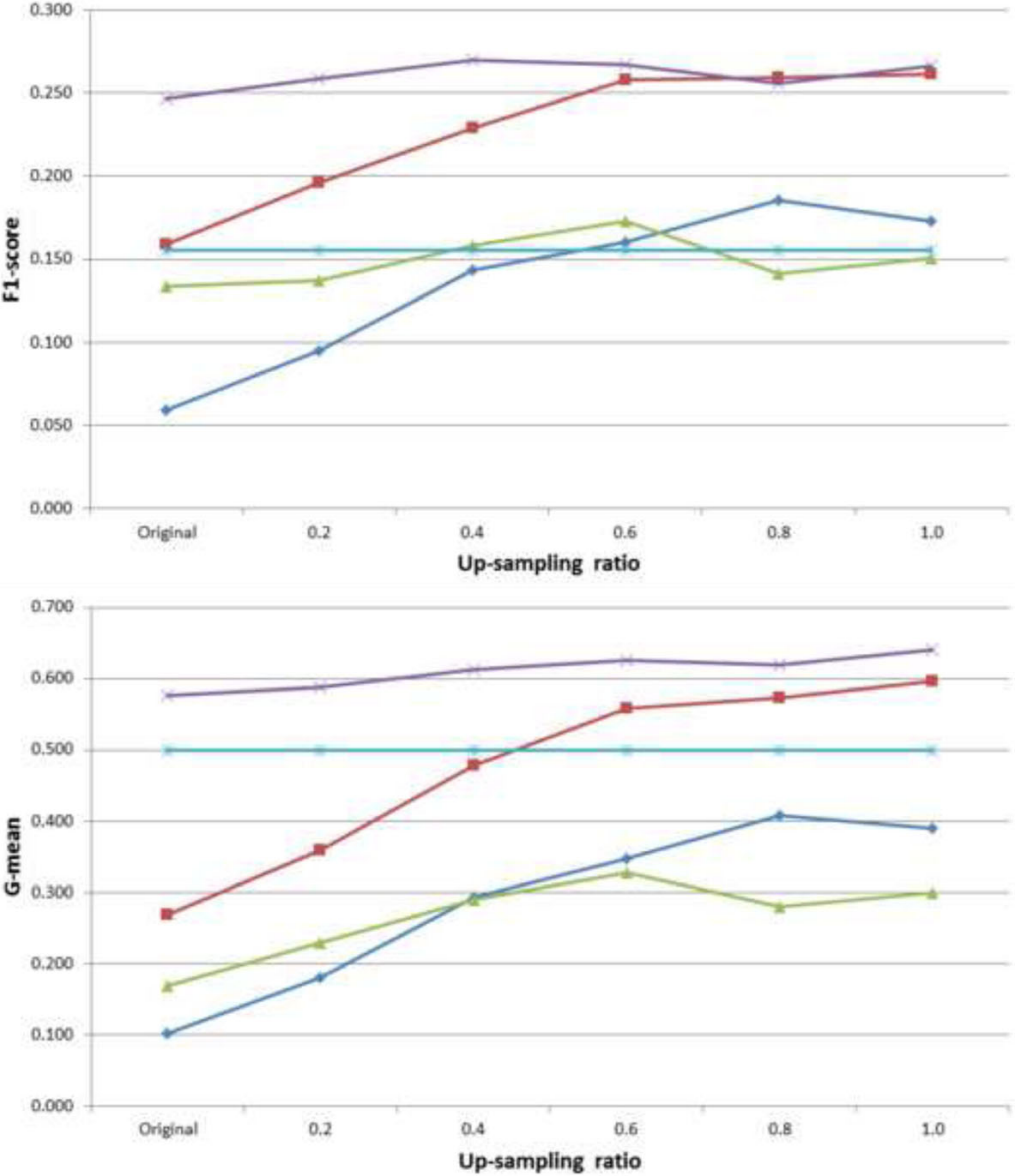
Average testing F1-scores (*top*) and G-means (*bottom*) from 20-run 10-fold cross-validation on Cardiac death = Yes, with original prevalence and different up-sampling ratios (x-axes). Legends: logistic regression (*blue diamonds*), naive Bayes (*red squares*), decision tree (*green triangles*), pattern discovery (*purple crosses*), and random baseline (*light blue asterisks*)

Figure 5 shows the extended up-sampling experiment results with respect to average testing F1-scores and G-means. Starting from up-sampling ratio 0.8, naive Bayes shows competitive F1-score (0.26 ± 0.02) to pattern discovery (0.26 ± 0.02). Pattern discovery remains consistent, with the lowest standard deviations (available in the Additional file 1), across all different ratios in both F1-scores and G-means. Consistently, the corresponding Wilcoxon test results on testing F1-scores and G-means are shown in Table 5. Note that pattern discovery is more convenient to interpret for domain users compared to naive Bayes.

**Table 5:** Wilcoxon test (paired, greater than) *p*-values between pattern discovery and the other methods on testing F1-scores and G-means of the cross validations on the cardiac death dataset

	Logistic Regression	Naive Bayes	Decision Tree
F-score			
Original	4.43 × 10^−5^	5.17 × 10^−5^	4.43 × 10^−5^
0.2	4.43 × 10^−5^	4.43 × 10^−5^	6.02 × 10^−5^
0.4	4.43 × 10^−5^	0.0012	9.45 × 10^−5^
0.6	4.43 × 10^−5^	0.1161	4.43 × 10^−5^
0.8	4.43 × 10^−5^	0.7492	4.43 × 10^−5^
1.0	4.43 × 10^−5^	0.1953	4.43 × 10^−5^
G-mean			
Original	4.43 × 10^−5^	4.43 × 10^−5^	4.43 × 10^−5^
0.2	4.43 × 10^−5^	4.43 × 10^−5^	4.43 × 10^−5^
0.4	4.43 × 10^−5^	4.43 × 10^−5^	4.43 × 10^−5^
0.6	4.43 × 10^−5^	4.43 × 10^−5^	4.43 × 10^−5^
0.8	4.43 × 10^−5^	4.43 × 10^−5^	4.43 × 10^−5^
1.0	4.43 × 10^−5^	1.27 × 10^−4^	4.43 × 10^−5^

*p-value = 4.43 × 10^−5^ indicates higher rankings of paired values in all 20 runs for pattern discovery

The experiment results on the cardiac death dataset demonstrate consistent robust prediction performance of pattern discovery, which reaches average testing F1-scores and G-means comparable to the best results achievable from various up-sampling ratios on training data. Furthermore, pattern discovery offers good interpretability for domain users, fitted best for our target scenario where initial insights are desired before potential formal follow-ups.

We illustrate the discovered pattern from full data and discuss its details in the Additional file 1. The interpretable pattern sheds light to predictive modeling of cardiac deaths before more data can be obtained, and can be used as screening reference for more in-depth follow-up and cohort studies for more detailed clinical and biological significance.

## Discussion

In this work we have targeted a practical scenario where domain users would like to perform first-hand prediction without requiring sophisticated handling on clinical data repositories with existing practice data, so that they can plan more precisely before more involving efforts are spent. On the two retrospective datasets, pattern discovery has shown promising results with good interpretability and competitive prediction performance without sophisticated data handling.

Pattern discovery is novel in its intuitively interpretable model combined with the optimized matching threshold to accommodate noise. Pattern discovery is designed for minority and noise challenges which association rule mining does not address. Different from non-interpretable methods (such as SVM, ANN), or impractically complex models (such as random forest, random tree), pattern discovery offers domain interpretability. It also shows competitive performance compared with representative interpretable methods including naive Bayes, logistic regression, and decision tree. Without sophisticated processing or tweaking (such as boosting and sampling techniques), pattern discovery can achieve predictive performance on imbalanced data comparable to the best achievable one.

As a good starting point for domain users to gain insights on clinical data repositories with existing practice data, pattern discovery can be further enhanced first into pattern visual analytics. With good interpretability, pattern discovery can be visualized and updated by users in an interactive manner. Clinical users can conveniently incorporate their knowledge into discovered patterns and check how the prediction performance will be influenced instantly. As a result, they are engaged to have a detailed understanding of both the predictive pattern and patient data, which can be utilized for follow-ups such as patient cohort design.

We are also aware of the limitation of this work for future improvement. We focus on comparisons among domain interpretable methods, and excluded methods which would provide stronger predictive performance by compromising interpretability. Our experiments were limited in the two retrospective dataset and rotated training-testing split was employed in cross-validation, but a real clinical application with training-testing split would better evaluate the actual predictive performance. Furthermore, the search/optimization towards optimal patterns will become more critical, especially with extensions to more advanced pattern modeling, such as auto-categorization for numeric attributes, multi-value and multi-pattern supports for better descriptive power.

## Conclusions

Pattern discovery has been developed with good interpretability and a simple but effective algorithm. On the two retrospective datasets with high imbalance ratios and noise where the other interpretable methods face difficulty without sophisticated technical data handling, pattern discovery has demonstrated to be robust and valuable for the minority target prediction. The prediction results and interpretable patterns can provide insights in an agile and inexpensive way for the potential formal studies. We are looking into several directions to further enhance the value of pattern discovery.

## Additional file

Additional file 1: Supplementary materials of the manuscript. (DOCX 68 kb)

## Abbreviations

ANN: Artificial neural network
BSI: Boosted SVM for imbalanced data
CAD: Coronary artery disease
CDR: Clinical data repository
CRF: Case report form
CVIS: Cardiovascular Information System
EMR: Electronic Medical Record
G-mean: Geometric mean of sensitivity and specificity
GUI: Graphical user interface
HIS: Healthcare Information System
LIS: Laboratory Information System
PCI: Percutaneous coronary intervention
pre: Precision
RIS: Radiology Information System
RUS: RUSBoost
sen: Sensitivity
SSVM: SVM + SMOTE
SVM: Support vector machine
UB: UnderBagging
