# Supplementary material for: Imbalanced target prediction with pattern discovery on clinical data repositories
Source: BMC Med Inform Decis Mak. 2017 Apr 20;17:47. doi: 10.1186/s12911-017-0443-3 (PMC5399417; doi:10.1186/s12911-017-0443-3)
Supplement: Supplementary file 1 — Supplementary materials of the manuscript. (DOCX 68 kb) [file 12911_2017_443_MOESM1_ESM.docx]

# Illustrative running examples

## The evaluation steps illustration

We take the illustrative pattern and evaluate it on the sample data from Table 1. The pattern matching results are illustrated in the following Table S1.

Table S1 An illustrative example of the CVIS patient data matching the pattern (matching threshold = 2/3)

| ID (not used in pattern) | PCI History | Hemoglobin | CRP | **Bleeding (T = t for Yes)** | Matched ratio | Pattern matched = TRUE |
| --- | --- | --- | --- | --- | --- | --- |
| 1 | **Yes** | **Abnormal** | **Abnormal** | **Yes** | 3/3 | **Yes** |
| 2 | No | **Abnormal** | **Abnormal** | No | 2/3 | *Yes* |
| 3 | No | Normal | N/A | No | 0/3 | No |
| 4 | **Yes** | Normal | Normal | No | 1/3 | No |
| 5 | **Yes** | **Abnormal** | N/A | **Yes** | 2/3 | **Yes** |
| 6 | No | Normal | Normal | No | 0/3 | No |

In this example, TP = 2 (samples of ID = 1, 5), where both the pattern is matched and T = t, FP = 1 (ID = 2), TN = 3 (ID = 3, 4, 6) and FN = 0. Therefore,

pre = 2/(2+1) = 2/3 = 0.67; sen = 2/(2+0) = 1; spec = 3/(3+1) = 0.75;

F1-score = 2*0.67*1/(0.67+1) = 0.80;

G-mean = $\sqrt{1*0.75}=$ 0.87

## The heuristic method illustration

To illustrate the heuristic method to select a value of an attribute, for the PCI History (Yes/No) with the target Bleeding = Yes (non-target: Bleeding = No), an illustrative table is shown to be generated as follows in Table S2:

Table S2 Illustrative counts for PCI history and Bleeding

|  | PCI History = Yes | PCI History = No |
| --- | --- | --- |
| Bleeding = Yes | 9 (45%) | 11 (55%) |
| Bleeding = No | 100 (10%) | 900 (90%) |
| Ratios (in own rows) | 45% vs 10% | 55% vs 90% |

In PCI History = Yes, the ratio of Bleeding = Yes (45%) is considerably higher than that in Bleeding = No (10%). Therefore, PCI History = Yes is still a potentially useful attribute value to distinguish the minority event Bleeding = Yes. Therefore, PCI History = Yes is associated with Bleeding = Yes. Similarly, PCI History = No is associated with Bleeding = No. In general, this method first calculates the row-wise ratios for each attribute value, and then scans each column to find the maximal vertical ratio from all rows, and associate the attribute value with that row.

In our experiments, this heuristic was more effective towards imbalanced targets than combination of individual attribute values maximizing the evaluation measure. This is intuitive in the sense that the combination of independent individual maxima is too greedy to be predictive for imbalanced targets.

# Attributes in the two published datasets

## Thoracic Dataset:

The original dataset contains 16 pre-operative attributes plus the one target attribute (Risk1Yr).

To simulate our target scenario, where first-hand analytics can be applied without much tweaking, we skipped the numeric attributes which are without well-established categorization. Therefore, PRE4: forced vital capacity (FVC), PRE5: forced expiration volume (FEV1), and AGE were skipped in our evaluation experiments. Instead PRE4 and PRE5 have known a combination rule for chronic obstructive pulmonary disease COPD = Yes / No. To provide categorical input, the transformation is as follows. Then it was transformed into a categorical attribute of chronic obstructive pulmonary disease (COPD = Yes if FEV1/FVC < 0.7; = No otherwise) with the auxiliary function, where a domain user could configure without programming.

Table S3 Attribute list of the thoracic dataset

| # | Attribute | Description (values) | Remark |
| --- | --- | --- | --- |
| 1 | DGN | Diagnosis - specific combination of ICD-10 codes for tumors if any (DGN3, DGN2, DGN4, DGN6, DGN5, DGN8, DGN1) |  |
| 2 | PRE4 | Forced vital capacity - FVC (numeric) | Used COPD; skipped |
| 3 | PRE5 | Volume that has been exhaled at the end of the first second of forced expiration - FEV1 (numeric) | Used COPD; skipped |
| 4 | PRE6 | Performance status - Zubrod scale (PRZ2, PRZ1, PRZ0) |  |
| 5 | PRE7 | Pain before surgery (T, F) |  |
| 6 | PRE8 | Haemoptysis before surgery (T, F) |  |
| 7 | PRE9 | Dyspnoea before surgery (T, F) |  |
| 8 | PRE10 | Cough before surgery (T, F) |  |
| 9 | PRE11 | Weakness before surgery (T, F) |  |
| 10 | PRE14 | T in clinical TNM - size of the original tumor, from OC11 (smallest) to OC14 (largest) (OC11, OC14, OC12, OC13) |  |
| 11 | PRE17 | Type 2 DM - diabetes mellitus (T, F) |  |
| 12 | PRE19 | MI up to 6 months (T, F) |  |
| 13 | PRE25 | PAD - peripheral arterial diseases (T, F) |  |
| 14 | PRE30 | Smoking (T, F) |  |
| 15 | PRE32 | Asthma (T, F) |  |
| 16 | AGE | Age at surgery (numeric) | Skipped |
| 17 | Risk1Y | 1 year survival period - (T)rue value if died (T,F) | Target |
| 18 | COPD | Chronic obstructive pulmonary disease Yes if FEV1/FVC < 0.7; No otherwise (Yes, No) | Derived |

## Cardiac Death Dataset:

The original dataset is composed of 43 attributes covering both clinical attributes and derived ones from gene expressions. The target attribute is Cardiac Death. There are 41 non-trivial attributes where patient ID was skipped, and neutrophils removed by the program with over 80% missing.

Table S4 Attribute list of the cardiac death dataset

| # | Attribute | Description (values) | Remark |
| --- | --- | --- | --- |
| 1 | ID | Patient ID | Skipped |
| 2 | GENDER | Gender (MAL, FEM) |  |
| 3 | BMI | BMI (numeric) | Categorized |
| 4 | CVD_TYPE | CVD Type (ACUTE, CAD, FINE, OLD) |  |
| 5 | AGE | Age (numeric) | Categorized |
| 6 | SystolicBP | Systolic Blood Pressure (numeric) | Categorized |
| 7 | DiastolicBP | Diastolic Blood Pressure (numeric) | Categorized |
| 8 | BMI3 | BMI3 (categorized BMI) | Skipped |
| 9 | DIABETES | Diabetes (Yes, No) |  |
| 10 | HTN | Hypertension (Yes, No) |  |
| 11 | LIPID | Lipid (Yes, No) |  |
| 12 | CAD | CAD (Yes, No) |  |
| 13 | Gensini | Gensini score (numeric) | Categorized |
| 14 | SMOKER | Smoker (current, former, never) |  |
| 15 | Statin | Statin (Yes, No) |  |
| 16 | ARB_ACE | ACE-inhibitor / ARB (Yes, No) |  |
| 17 | Aspirin | Aspirin (Yes, No) |  |
| 18 | BB | Beta blocker (Yes, No) |  |
| 19 | Plavix | Plavix (clopidogrel) (Yes, No) |  |
| 20 | HDL | HDL cholesterol (numeric) | Categorized |
| 21 | TRIG | Triglyceride (numeric) | Categorized |
| 22 | LDL | LDL cholesterol (numeric) | Categorized |
| 23 | CHOL | Total cholesterol (numeric) |  |
| 24 | GLU | Glucose (numeric) | Categorized |
| 25 | CREAT | Serum Creatinine (numeric) | Categorized |
| 26 | WBC | White Blood Cell (numeric) | Categorized |
| 27 | Neutrophils | Neutrophils (numeric) | Skipped; missing >80% |
| 28-37 | snmAxis* | 1-10 for 10 derived axes of variations from gene expression (numeric) | Categorized |
| 38-42 | Prin* | 1-5 for 5 principal components derived from gene expression (numeric) | Categorized |
| 43 | Cardiac Death | Cardiovascular death during follow up (Yes, No) | Target |
| 44 | Blood pressure | Normal if both systolic and diastolic blood pressures are normal; otherwise Abnormal | Derived |

For the many numeric attributes, fortunately there are well established categorizations in the domain for normal ranges without tweaking requirements. By utilizing the auxiliary categorization function, existing domain knowledge is conveniently incorporated to provide easily interpretable categorized attributes. The known normal ranges that were automatically categorized into Normal by the auxiliary function are listed below. Values beyond the ranges were categorized into Abnormal.

Table S5 Attribute categorization using the known normal range

| Attribute | Normal range |
| --- | --- |
| BMI | [20, 25] |
| Systolic Blood Pressure | [90, 140] |
| Diastolic Blood Pressure | [60, 90] |
| Gensini score | <= 25 |
| HDL cholesterol | >= 40 |
| LDL cholesterol | <= 120 |
| Triglyceride | <= 150 |
| Total cholesterol | <= 200 |
| Glucose | [70, 125] |
| Serum Creatinine | [0.5, 1.2] |
| White Blood Cell | [4, 10] |
| Age | <= 80 |

For the gene expression related attributes, we simply used the signs (> 0 or ≤ 0) to follow the minimal tweaking scenario. Further exploration on categorizing the numeric gene expressions definitely could reveal better predictability, but we stay focused on interpretability with minimal knowledge on the unestablished expression categorization.

# Evaluation result details on both datasets

Evaluation results on F1-score and G-mean with their standard deviations (±) are shown in the following tables. The best performance and lowest standard deviations are shown in bold.

Table S6 F1-score, G-mean results with the standard deviations for the thoracic dataset

| F1-score | Original | 0.2 | 0.4 | 0.6 | 0.8 | 1.0 |
| --- | --- | --- | --- | --- | --- | --- |
| Logistic Regression | 0.058 | 0.082 | 0.237 | 0.282 | 0.318 | 0.311 |
| Naive Bayes | 0.092 | 0.109 | 0.229 | 0.297 | 0.327 | 0.337 |
| Decision Tree | 0.001 | 0.011 | 0.164 | 0.216 | 0.224 | 0.211 |
| Pattern Discovery | **0.304** | **0.310** | **0.313** | **0.326** | **0.340** | **0.345** |
| Random Baseline | 0.230 | 0.230 | 0.230 | 0.230 | 0.230 | 0.230 |

| F1 ± standard deviation | Original | 0.2 | 0.4 | 0.6 | 0.8 | 1.0 |
| --- | --- | --- | --- | --- | --- | --- |
| Logistic Regression | 0.021 | 0.024 | 0.031 | 0.036 | 0.025 | 0.019 |
| Naive Bayes | 0.020 | 0.032 | 0.029 | 0.025 | 0.020 | 0.020 |
| Decision Tree | **0.005** | 0.017 | 0.040 | 0.028 | 0.028 | 0.019 |
| Pattern Discovery | 0.011 | **0.012** | **0.015** | **0.014** | **0.013** | **0.010** |

| G-mean | Original | 0.2 | 0.4 | 0.6 | 0.8 | 1.0 |
| --- | --- | --- | --- | --- | --- | --- |
| Logistic Regression | 0.098 | 0.140 | 0.388 | 0.490 | 0.564 | 0.575 |
| Naive Bayes | 0.158 | 0.185 | 0.400 | 0.523 | 0.587 | 0.616 |
| Decision Tree | 0.002 | 0.018 | 0.293 | 0.390 | 0.428 | 0.439 |
| Pattern Discovery | **0.583** | **0.592** | **0.594** | **0.607** | **0.626** | **0.633** |
| Random Baseline | 0.500 | 0.500 | 0.500 | 0.500 | 0.500 | 0.500 |

| G-mean ± standard deviation | Original | 0.2 | 0.4 | 0.6 | 0.8 | 1.0 |
| --- | --- | --- | --- | --- | --- | --- |
| Logistic Regression | 0.036 | 0.040 | 0.039 | 0.039 | 0.025 | 0.025 |
| Naive Bayes | 0.038 | 0.051 | 0.044 | 0.031 | 0.022 | 0.023 |
| Decision Tree | **0.008** | 0.027 | 0.056 | 0.041 | 0.044 | 0.033 |
| Pattern Discovery | 0.014 | **0.014** | **0.017** | **0.018** | **0.016** | **0.011** |

Table S7 F1-score, G-mean results with the standard deviations for the cardiac death dataset

| F1-score | Original | 0.2 | 0.4 | 0.6 | 0.8 | 1.0 |
| --- | --- | --- | --- | --- | --- | --- |
| Logistic Regression | 0.059 | 0.095 | 0.143 | 0.160 | 0.185 | 0.173 |
| Naive Bayes | 0.158 | 0.196 | 0.229 | 0.258 | **0.259** | 0.262 |
| Decision Tree | 0.134 | 0.137 | 0.158 | 0.172 | 0.141 | 0.150 |
| Pattern Discovery | **0.247** | **0.259** | **0.270** | **0.267** | 0.256 | **0.266** |
| Random Baseline | 0.155 | 0.155 | 0.155 | 0.155 | 0.155 | 0.155 |

| F1 ± standard deviation | Original | 0.2 | 0.4 | 0.6 | 0.8 | 1.0 |
| --- | --- | --- | --- | --- | --- | --- |
| Logistic Regression | 0.032 | 0.041 | 0.038 | 0.029 | 0.029 | 0.037 |
| Naive Bayes | 0.039 | 0.035 | 0.038 | 0.029 | 0.018 | 0.033 |
| Decision Tree | 0.056 | 0.061 | 0.066 | 0.043 | 0.047 | 0.051 |
| Pattern Discovery | **0.025** | **0.024** | **0.023** | **0.020** | **0.017** | **0.023** |

| G-mean | Original | 0.2 | 0.4 | 0.6 | 0.8 | 1.0 |
| --- | --- | --- | --- | --- | --- | --- |
| Logistic Regression | 0.102 | 0.181 | 0.294 | 0.347 | 0.408 | 0.389 |
| Naive Bayes | 0.269 | 0.360 | 0.478 | 0.560 | 0.573 | 0.597 |
| Decision Tree | 0.168 | 0.229 | 0.290 | 0.328 | 0.281 | 0.300 |
| Pattern Discovery | **0.577** | **0.588** | **0.612** | **0.627** | **0.620** | **0.641** |
| Random Baseline | 0.500 | 0.500 | 0.500 | 0.500 | 0.500 | 0.500 |

| G-mean ± standard deviation | Original | 0.2 | 0.4 | 0.6 | 0.8 | 1.0 |
| --- | --- | --- | --- | --- | --- | --- |
| Logistic Regression | 0.056 | 0.072 | 0.071 | 0.050 | 0.057 | 0.067 |
| Naive Bayes | 0.062 | 0.060 | 0.069 | 0.049 | 0.033 | 0.056 |
| Decision Tree | 0.069 | 0.094 | 0.105 | 0.076 | 0.084 | 0.087 |
| Pattern Discovery | **0.048** | **0.048** | **0.043** | **0.033** | **0.028** | **0.036** |

# Evaluation results on up-sampling ratios beyond 1.0 on the thoracic dataset

Although it was no long valid for the imbalance assumption, we further investigated into the performance with up-sampling ratios beyond 1.0. As illustrated in Figure S1 with testing F1-scores, further up-sampling in training does not always help to improve the testing performance. Pattern discovery still maintains robust performance (F1-scores no less than 0.3). Decision tree reaches its top F1-score 0.25 at ratio 0.2, compared to 0.211 at ratio 0.1 which is below baseline. G-means also show the same conclusion (details not shown) where pattern discovery maintains its robust performance. The degeneration of logistic regression and naïve Bayes is even more drastic (G-means below baseline when up-sampling ratios > 2.0) while G-means of pattern discovery are no less than 0.58.

Figure S1. Average testing F1-scores and G-means (y-axes) from 20-run 10-fold cross-validation on Risk1Yr = Yes of the thoracic dataset, with different up-sampling ratios (x-axes).


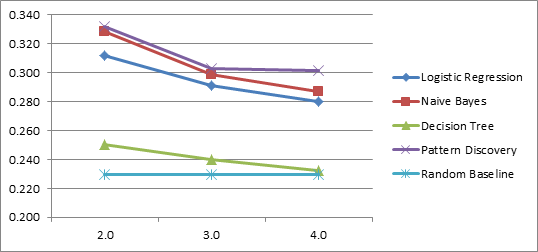


# JRip+BSI rules on the thoracic dataset

Table S8 JRip+BSI rules from reference

| Rule | Coverage | Accuracy |
| --- | --- | --- |
| (DGN = DGN5) => Risk1Yr = T | 0.03 | 0.47 |
| (PRE14 = OC14) => Risk1Yr = T | 0.04 | 0.41 |
| (PRE17 = T) and (PRE30 = T) and (AGE > = 57) =>Risk1Yr=T | 0.05 | 0.38 |
| (PRE11 = T) and (PRE5 < = 2.16) and (PRE4 > = 2.44) =>Risk1Yr = T | 0.05 | 0.35 |
| (PRE9 = T) and (AGE > = 54) and (PRE5 < = 66.4) => Risk1Yr = T | 0.05 | 0.35 |
| (PRE14 = OC13) => Risk1Yr = T | 0.04 | 0.32 |
| (DGN = DGN2) and (PRE30 = T) and (PRE14 = OC12) and (PRE5 < = 3.72) => Risk1Yr = T | 0.04 | 0.3 |
| (PRE8 = T) and (PRE30 = T) and (PRE4 < = 3.52) => Risk1Yr = T | 0.08 | 0.26 |
| OTHERWISE => Risk1Yr = F | 0.62 | 0.97 |

# Discovered pattern on the cardiac dataset

For illustration, we show the pattern discovered with all data on the top 10 significant attributes for cardiac death = Yes as follows:

Table S9 Discovered pattern from full cardiac dataset for illustration

| Pattern (Rule) |
| --- |
| Age = Over 80, snmAxis8 = Negative, Gensini score = Abnormal (>25), Prin3 = Positive, Hypertension = Yes, Serum Creatinine = Abnormal (beyond [0.5, 1.2]), snmAxis1 = Positive, Triglyceride = Normal (within 0:150), Prin4 = Negative, BMI = Normal (20-25); *r* = 60% -> cardiac death = Yes |
| OTHERWISE -> cardiac death = No |

Note that Prin* and snmAxis* indicate principal components and conserved axes of variation respectively of the gene expressions. On all data this pattern achieves F1-score 0.404, compared to 0.304 and 0.158 of naive Bayes and logistic regression, respectively. Note that attribute-values in the pattern work collectively (not individually) to reach the prediction performance.

Significant attributes age (p = 7.1e-5), hypertension (p = 0.02) and serum creatinine (0.03) are consistent with the association study of the data^5^, further identified with reasonable values in the pattern. While our sign categorization on expression attributes could be oversimplified (beyond our focus scope), the pattern is able to get a combination of snmAxis8, snmAxis1, Prin3 and Prin4, where snmAxis1 and Prin3 were highlighted for their potential functional meanings in the previous study. Interestingly, Gensini score and triglyceride are present in the pattern. Gensini quantifies CAD severity, and high triglyceride is linked to atherosclerosis, and how it relates to HDL-cholesterol associated to CAD as well as cardiac death could be further investigated. The combination suggests the synergistic potential of using both clinical and gene expression characteristics for cardiac death prediction.
